# Short-Term Biceps Muscle Wasting Assessed by Serial Ultrasound as a Predictor of Survival Duration in Terminally Ill Cancer Patients: A Retrospective Cohort Study

**DOI:** 10.3390/medicina62020292

**Published:** 2026-02-01

**Authors:** İrem Kıraç Utku, Nezahat Müge Çatıkkaş, Deniz Sevindik Günay, Ayfer Durak, Burcu Gülbağcı, Umut Safer

**Affiliations:** 1 Division of Geriatrics, Department of Internal Medicine, Tekirdag Ismail Fehmi Cumalioglu City Hospital, Tekirdag 59030, Turkey; 2 Division of Geriatrics, Department of Internal Medicine, Sancaktepe Martyr Prof. Dr. Ilhan Varank Training and Research Hospital, Istanbul 34785, Turkey; 3 Division of Geriatrics, Department of Internal Medicine, Fatih Sultan Mehmet Training and Research Hospital, Istanbul 34752, Turkey; 4 Division of Geriatrics, Department of Internal Medicine, Amasya Sabuncuoglu Serefeddin Training and Research Hospital, Amasya 05100, Turkey; 5 Division of Oncology, Department of Internal Medicine, Tekirdag Ismail Fehmi Cumalioglu City Hospital, Tekirdag 59030, Turkey; burcubln@gmail.com

**Keywords:** sarcopenia, ultrasonography, palliative care, terminal care, muscle weakness

## Abstract

*Background and Objectives:* Rapid physiological decline in terminal cancer is frequently accompanied by accelerated skeletal muscle loss. Although bedside ultrasonography (US) is practical and feasible in palliative care settings, the prognostic relevance of short-term muscle change remains unclear. This study aimed to evaluate whether the rate of muscle loss over a 10-day period, assessed by serial ultrasound, is associated with survival duration in terminally ill cancer patients. *Materials and Methods:* This single-center retrospective cohort study included 87 inpatients with end-stage cancer who underwent bedside ultrasound measurements of the biceps brachii (BB) and rectus femoris (RF). Baseline US was performed within the first three days of admission, followed by a repeat assessment 10 days after baseline (day-10 follow-up ultrasound). Muscle thickness (MT) measurements were normalized by height squared (m^2^), and 10-day changes were calculated as delta (Δ) indices, defined as baseline minus day-10 values. Because the exposure of interest (Δ) can only be determined after completion of the day-10 assessment, survival timing analyses were anchored to this prespecified landmark. Survival duration was defined as the number of days from the day-10 follow-up ultrasound to death among patients who died within one year. Associations between muscle changes and survival duration were evaluated using correlation analyses and multivariable linear regression adjusted for age, sex, body mass index, Eastern Cooperative Oncology Group (ECOG) performance status, and nutritional risk. The primary analyses focused on survival timing among decedents. *Results:* Significant muscle loss was observed over the 10-day interval between baseline and 10 days after baseline. Among the 58 patients who died within one year, greater short-term biceps muscle loss, reflected by higher Δ BB muscle thickness index (Δ BB MT-I), was moderately associated with shorter survival duration (r = −0.437, *p* = 0.0006). In multivariable linear regression analysis, Δ BB MT-I remained independently associated with survival duration (β = −701.19; 95% CI: −1102 to −301; *p* = 0.0006), whereas RF muscle changes and baseline clinical variables were not statistically significant. *Conclusions:* Short-term biceps muscle loss assessed by serial ultrasound, as reflected by Δ BB MT-I, is associated with shorter survival duration in terminally ill cancer patients. These findings suggest that dynamic muscle changes, rather than single-time-point measurements, may provide clinically meaningful insight into short-term survival timing. Serial bedside muscle ultrasound may serve as a low-burden adjunct for prognostic communication in palliative care, although prospective time-to-event studies are required for validation.

## 1. Introduction

In terminally ill patients with malignancy, chronic inflammation, malnutrition, and metabolic disturbances related to both the tumor itself and its treatment may accelerate muscle proteolysis, resulting in a marked reduction in skeletal muscle mass, including muscle atrophy, sarcopenia, and cachexia [1,2]. This loss of muscle mass not only leads to reduced physical strength and functional impairment but may also adversely affect treatment tolerance, quality of life, and survival [1,3].

Commonly used methods for assessing muscle mass—such as computed tomography (CT), dual-energy X-ray absorptiometry (DEXA), bioelectrical impedance analysis (BIA), and anthropometry—provide quantitative information but have important limitations. In bedridden, terminally ill inpatients, CT requires patient transport, involves radiation exposure, is costly, and is difficult to repeat; DEXA has limited accessibility; and BIA and anthropometric measures may be substantially influenced by hydration status, edema, or acute physiological disturbances. These factors limit their practicality in advanced palliative care settings [4,5].

In contrast, muscle ultrasonography offers a non-invasive, radiation-free, portable, bedside-applicable, and repeatable assessment method, making it particularly suitable for frail and immobile patients. Most existing ultrasound studies in oncology and geriatrics have focused on single-time-point muscle measurements, which have been associated with outcomes such as frailty, functional decline, and mortality [6,7,8]. However, static assessments provide limited insight into the rate of biological deterioration, and evidence evaluating short-interval serial changes in muscle mass assessed by ultrasound and their association with survival outcomes remains extremely limited, particularly in palliative care populations.

Moreover, commonly used single-time-point clinical indicators in terminally ill patients—such as age, body mass index (BMI), ECOG performance status, or nutritional risk scores—are insufficient for predicting the timing of death and fail to capture the dynamic pace of decline near the end of life [9,10]. In end-stage cancer patients, predicting not only whether death will occur but also when it is likely to occur is critically important for prognostic discussions, care planning, timing of supportive interventions, and communication with patients and family members [11,12]. In this context, short-term muscle loss may serve as a surrogate marker of accelerated biological deterioration rather than cumulative disease burden, reflecting the pace of systemic decline in advanced cancer [13]. As such, it may provide clinically meaningful prognostic information primarily for communication and care planning purposes rather than for therapeutic risk stratification [11].

Supporting this concept, serial CT-based studies have demonstrated that muscle loss accelerates as patients approach the end of life. For example, Lee et al. reported a marked acceleration in skeletal muscle area loss at the L1 level during the final three months of life in patients with advanced lung cancer, with faster muscle loss associated with shorter survival [14]. Similar observations from longitudinal CT-based body composition studies further support the concept that late-stage muscle depletion reflects a severe catabolic state closely linked to mortality rather than baseline disease burden [15,16].

Building on this background, the present study aims to contribute to this underexplored area by evaluating short-term muscle loss using serial ultrasound-based measurements of the biceps brachii (BB) and rectus femoris (RF) obtained within the first 10 days of hospitalization in terminal palliative care inpatients. To reduce bias related to interindividual anthropometric differences, muscle thickness measurements were normalized by height squared (m^2^) to generate muscle thickness indices, and short-term temporal changes (Δ indices) were analyzed. This approach enables a more comparable assessment of the dynamic rate of muscle depletion across individuals and is consistent with contemporary frameworks for evaluating muscle quantity in advanced disease states [13,17].

Although interest in ultrasound-based muscle assessment has increased, studies examining serial ultrasound-derived muscle loss and mortality have largely been conducted in intensive care unit (ICU) populations. Prior ICU studies have consistently shown that rapid muscle wasting assessed by serial ultrasonography is associated with adverse outcomes, including prolonged mechanical ventilation, functional impairment, and increased mortality [18,19]. These findings highlight the prognostic relevance of short-term dynamic muscle loss in critically ill patients.

In contrast, evidence regarding whether similar short-term dynamic muscle changes carry prognostic significance in terminal palliative cancer patients remains scarce. Available data in this population are limited to a small number of cross-sectional or short-term observational studies, which primarily rely on single-time-point ultrasound measurements rather than serial assessments [20,21]. As a result, the prognostic value of short-interval ultrasound-derived muscle loss in terminal palliative care settings remains insufficiently explored.

Furthermore, differences between upper- and lower-limb muscle groups may be particularly relevant in bedridden patients. Upper-limb muscles such as the biceps brachii (BB) may retain intermittent activity related to positioning, feeding, transfers, or assisted care, whereas lower-limb muscles such as the quadriceps are more profoundly affected by immobilization and disuse, particularly in bedridden patients [22]. Consistent with this notion, experimental and clinical studies indicate that lower-limb muscles are especially vulnerable to disuse-related atrophy, while upper-limb muscles may better reflect residual functional reserve [23,24]. These differing patterns of loading and disuse may therefore influence both the rate and prognostic relevance of muscle loss, providing a biological rationale for evaluating these muscle groups separately.

Based on this rationale, we hypothesized that greater short-term loss in the biceps brachii muscle thickness index (Δ BB MT-I), defined as baseline minus day-10 values, would be associated with shorter survival duration. Accordingly, we aimed to evaluate the association between Δ BB MT-I measured at admission and at day 10 and the time from the fixed day-10 follow-up ultrasound landmark to death in terminally ill cancer patients receiving palliative care. Rectus femoris-based measures and cross-sectional area indices were additionally assessed as exploratory analyses.

## 2. Materials and Methods

This study was designed as a single-center retrospective cohort investigation. Clinical and ultrasound data from terminally ill patients with malignancy who were hospitalized in the palliative care unit of the University of Health Sciences Sancaktepe Şehit Prof. Dr. İlhan Varank Training and Research Hospital between 1 January 2024 and 1 June 2024 were evaluated. Muscle ultrasonography was performed routinely as part of standard clinical care according to unit protocol. The baseline examination was conducted on the day of admission or within the first three days of hospitalization; the follow-up ultrasound was scheduled 10 days after the baseline examination (day-10 follow-up).

This study is based on a retrospective analysis of real-life clinical practice data derived from these routine measurements. Patients were eligible for inclusion if they had a diagnosis of terminal-stage malignancy, had discontinued oncologic treatment, and were hospitalized in the palliative care unit. Inclusion additionally required the availability of muscle ultrasonography measurements performed at baseline and at the day-10 follow-up assessment, as well as recorded data on height, body weight, ECOG performance status, NRS-2002 score, and malignancy type.

Patients were excluded if they died, were discharged, or were transferred before completion of the 10-day ultrasound follow-up cycle. Additional exclusion criteria included deformity, amputation, or casting of the upper or lower extremities that precluded reliable ultrasound measurement, as well as inability to clearly delineate muscle fascial borders due to extensive edema or anasarca.

A total of 87 patients meeting these criteria were included in the final analysis.

### 2.1. Muscle Ultrasonography

Muscle ultrasonography was performed at baseline within the first three days after admission. Most patients underwent the initial assessment on day 1 (74/87, 85.1%), followed by day 2 (9/87, 10.3%) and day 3 (4/87, 4.6%). Follow-up ultrasonography was performed 10 days after the baseline examination (hereafter referred to as the day-10 follow-up). All measurements were conducted bedside by the same experienced geriatrician, thereby minimizing observer-dependent variability.

All ultrasound examinations were performed using a Philips Affiniti 50 ultrasound system (Philips Healthcare, Bothell, WA, USA) equipped with a 7.5 MHz, 5 cm linear probe. B-mode imaging was used with patients in the supine position and the extremities relaxed. Minimal probe pressure was applied to avoid tissue compression. Each measurement was repeated three times, and the mean value was recorded for analysis.

Rectus femoris (RF) measurements were obtained at the distal one-third point of the distance between the anterior inferior iliac spine and the superior border of the patella, whereas biceps brachii (BB) measurements were performed at the midpoint between the acromion and the olecranon. For each muscle, subcutaneous fat thickness (SFTT), muscle thickness (MT), and cross-sectional area (CSA) were assessed. Image selection was based on the highest-quality view with clearly defined fascial borders and absence of imaging artifacts.

Due to the retrospective nature of the study, intraclass correlation coefficients (ICCs) could not be calculated within this cohort. However, intraobserver reliability of ultrasound-based muscle measurements performed by the same operator has been previously evaluated in an independent dataset using a repeated-measurement protocol. In that validation analysis, ICCs were calculated using two images obtained at 15-min intervals from 15 healthy participants, demonstrating excellent reliability, with ICC values of 0.98, 0.86, and 0.93 for RF SFTT, MT, and CSA, and 0.97, 0.95, and 0.99 for BB SFTT, MT, and CSA, respectively [8].

### 2.2. Muscle Index and Delta Calculations

Muscle thickness (MT) measurements were normalized by height squared (m^2^) to reduce interindividual anthropometric variability and to improve the comparability of short-term muscle loss across patients with different body sizes. Although height squared (m^2^) normalization is more commonly applied to area-based muscle indices, this body-size adjustment principle was intentionally extended to ultrasound-derived MT measurements to standardize the scale of Δ values in population-level regression analyses. This approach is consistent with established sarcopenia frameworks, including the European Working Group on Sarcopenia in Older People (EWGSOP) and the Asian Working Group for Sarcopenia (AWGS), which recommend body-size-adjusted assessment of muscle quantity [25,26]. In addition, precedent exists in the point-of-care ultrasound literature, where thickness-based muscle indices normalized by height squared (m^2^) have been applied [27,28].

Δ values were calculated as baseline minus day-10 measurements (Δ = baseline − day-10), such that positive Δ values indicate short-term muscle loss over the 10-day interval.

### 2.3. Demographic and Clinical Data

Age, sex, BMI, ECOG performance status, NRS-2002 scores, and malignancy types were retrieved from the electronic medical record system.

### 2.4. Mortality Outcomes

Two outcomes were assessed: (1) one-year mortality (alive vs. deceased) in the full cohort (n = 87) and (2) post-day-10 time to death (death timing). For the death-timing outcome, the prespecified day-10 follow-up ultrasound was used as a landmark time point because Δ values can only be calculated after completion of the day-10 assessment. Accordingly, time to death was defined as the number of days from the day-10 follow-up ultrasound to death. Death dates were verified using both the hospital electronic medical record system and the national MERNIS registry.

Death-timing analyses were restricted to patients with an observed death within one year (n = 58), for whom an exact post-day-10 time-to-death could be determined. Patients who were alive at one year (n = 29) were included in descriptive and between-group analyses but were not eligible for the decedents-only time-to-death regression because an event time was not observed within the one-year follow-up window. As a supportive sensitivity analysis, an exploratory landmark Cox proportional hazards model was additionally performed in the full cohort, with day 10 defined as time zero and one-year survivors censored at 365 days.

### 2.5. Statistical Analysis

Statistical analyses were performed using IBM SPSS Statistics 26.0 (IBM Corp., Armonk, NY, USA). The distribution characteristics of continuous variables were assessed using the Shapiro–Wilk test; normally distributed data were reported as mean ± standard deviation, and non-normally distributed data as median (IQR). Categorical variables were presented as number and percentage (%). Equality of variances for normally distributed variables was verified using Levene’s test. For comparisons between two groups, Student’s *t*-test was used when parametric assumptions were met, and the Mann–Whitney U test was used otherwise.

The association between 10-day changes in muscle indices (Δ) and survival duration was primarily evaluated using Pearson correlation analysis, as the variables were continuous and scatterplots demonstrated an approximately linear trend; additionally, Spearman correlation was reported as a supportive analysis. Positive Δ values indicate short-term muscle loss. A multivariable linear regression model was constructed to identify independent determinants of survival duration. Covariates included in the model (age, sex, BMI, ECOG, and NRS-2002 scores) were selected based on clinical relevance and literature support, thereby preventing statistical over-adjustment. Regression assumptions—normality, homoscedasticity, and linearity—were tested using both graphical and statistical methods.

*p*-values obtained from multidimensional comparisons of muscle parameters were reported without correction due to the exploratory nature of the analyses and were interpreted for hypothesis generation. To reduce the potential impact of outliers, robust regression with a Huber M-estimator was applied as an additional analysis, demonstrating preservation of the direction and significance of the correlation observed in the primary analysis.

A post-hoc power analysis was performed for the study’s primary inference, the linear relationship between Δ BB MT-I and survival duration. Based on the observed correlation coefficient (r = –0.44, n = 58, two-tailed α = 0.05), the statistical power to detect this association was calculated as approximately 95%, indicating that the sample size was adequate to identify a medium-to-large effect. A *p*-value < 0.05 was considered statistically significant for all tests.

As a supportive sensitivity analysis, an exploratory landmark Cox proportional hazards model was performed with the day-10 follow-up ultrasound defined as time zero, with patients who were alive at one year censored at 365 days.

### 2.6. Ethical Approval

The study protocol was approved by the Clinical Research Ethics Committee of the University of Health Sciences Sancaktepe Şehit Prof. Dr. İlhan Varank Training and Research Hospital (Decision No: 2025/412). The study was retrospective in nature and conducted in accordance with the principles of the Declaration of Helsinki.

## 3. Results

A total of 87 terminally ill patients followed in the palliative care unit were included in the study. The mean age of the patients was 74.6 ± 10.2 years, and 54% were male. Most patients had severe functional impairment; ECOG performance levels 2–4 accounted for 86.2% of the cohort. Regarding BMI distribution, 19.5% of the patients were underweight, 55.2% were normal weight, 18.4% were overweight, and 6.9% were obese. Evaluation of NRS scores showed that 6.9% had NRS = 3, 18.4% had NRS = 4, 42.5% had NRS = 5, and 32.2% had NRS = 6. It was observed that 66.7% of the patients died within one year (Table 1).

Normalized ultrasound measurement results are summarized in Table 2. A consistent decline in both RF and BB muscle indices was observed over the 10-day interval between the baseline and follow-up ultrasound assessments (Table 2 and Table 3).

Across the cohort, serial ultrasound measurements demonstrated a marked reduction in muscle indices over the 10-day interval, indicating rapid and substantial short-term muscle loss in terminal palliative care patients. This short-term decline was most pronounced in the BB MT-I and BB CSA-I, suggesting that upper-extremity muscle reserves may be particularly vulnerable during the terminal stage (Table 3).

Baseline clinical variables (age, sex, BMI, ECOG, NRS-2002) did not differ significantly between patients who died within one year and those who survived (all *p* > 0.17). Muscle delta values also showed no significant differences between the two mortality outcome groups (all *p* > 0.05) (Table 4).

When examining associations with survival duration, the strongest correlation was observed for Δ BB MT-I (Pearson r = −0.437, *p* = 0.0006), whereas the associations for the other muscle indices were weaker and did not reach statistical significance (Table 5).

In multivariable linear regression analysis, Δ BB MT-I was the only variable that remained independently associated with survival duration (β = −701.19, SE = 204.24, *p* = 0.0006), whereas age, sex, ECOG performance status, BMI, and NRS-2002 score were not statistically significant (Table 6). At the population level, the regression coefficient indicates that higher Δ BB MT-I values (reflecting greater short-term muscle loss) over the 10-day interval were associated with a shorter survival duration measured from the day-10 follow-up ultrasound.

**Figure 1:**
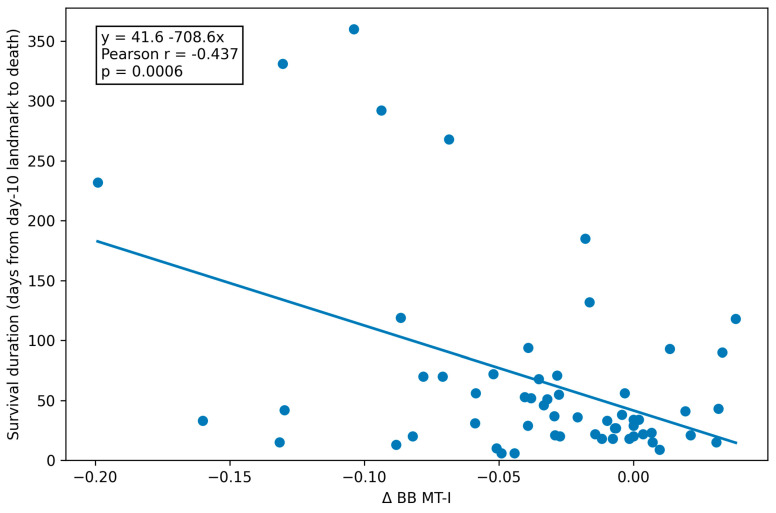
Association Between Δ BB MT-I and Survival Duration (days from the day-10 follow-up ultrasound to death) (n = 58). Survival duration was defined as the number of days from the prespecified day-10 follow-up ultrasound landmark to death among patients who died within one year (n = 58). The solid line represents the fitted unadjusted linear regression model. The fitted regression equation was: survival duration (days) = 41.6 − 708.6 × (Δ BB MT-I). Pearson correlation and regression analyses indicate a statistically significant linear association between Δ BB MT-I and survival duration (Pearson r = −0.437; 95% CI −0.625 to −0.202; *p* = 0.0006).

The scatterplot demonstrated a clear linear association between Δ BB MT-I and survival duration (Figure 1). The regression line shown in Figure 1 represents the unadjusted (univariable) linear relationship between Δ BB MT-I and survival duration, whereas the estimates presented in Table 6 are derived from a multivariable-adjusted model. The univariable model explained 23% of the variance in survival duration (R^2^ = 0.227). The robustness of this association and its independence from outlier influence were further supported by Huber-weighted robust regression analysis (Figure 1).

To address potential survivor or immortal-time bias introduced by the requirement of completing the 10-day ultrasound assessment, an exploratory landmark time-to-event analysis with day 10 defined as time zero was performed. In this Cox proportional hazards model with censoring of survivors, Δ BB MT-I was associated with post-day-10 mortality hazard (HR 1.60 per 0.10-unit increase; 95% CI 1.04–2.44; *p* = 0.033), whereas no other covariates reached statistical significance. These results are presented in Supplementary Table S1.

In a sensitivity analysis adjusting for the timing of the baseline ultrasound examination (day 1–3 after admission), the association between Δ BB MT-I and survival duration remained materially unchanged (Supplementary Table S2).

## 4. Discussion

This study is among the limited number of investigations that quantitatively evaluate short-term muscle loss using serial muscle ultrasonography in terminally ill patients with malignancy and examine its association with survival duration. Our findings demonstrate that significant atrophy develops in both the rectus femoris (RF) and biceps brachii (BB) muscles within the first 10 days of hospitalization. However, the strongest association with survival duration was observed for short-term biceps muscle loss assessed by the Δ biceps brachii muscle thickness index (Δ BB MT-I).

Importantly, Δ BB MT-I values did not differ significantly between patients who died within one year and those who survived beyond one year. Nevertheless, among patients who died within one year, greater short-term biceps muscle loss was associated with a shorter interval between the day-10 follow-up ultrasound and death. These findings suggest that Δ BB MT-I may reflect the timing or proximity of death rather than serving as a determinant of whether death occurs. To our knowledge, this is among the first studies to examine the association between short-term ultrasound-derived Δ muscle indices and survival timing in terminal cancer patients.

The relationship between muscle mass, sarcopenia, and mortality has been extensively discussed in the literature, and both the European and Asian consensus statements on sarcopenia cite numerous studies demonstrating associations with mortality, hospitalization, and functional decline [25,26]. However, most of these investigations rely on single-time-point measurements and predominantly use computed tomography, dual-energy X-ray absorptiometry, or bioimpedance-based assessments of muscle mass [29]. Although these modalities provide detailed information on muscle quantity and composition, their routine use in terminal palliative patients is often limited by radiation exposure, the need for patient transport, cost, and reduced feasibility.

In this context, ultrasonography offers several practical advantages, including the absence of radiation exposure, bedside applicability, and suitability for repeated assessments in bedridden patients [30]. Recent systematic reviews and meta-analyses have further demonstrated that ultrasound shows acceptable diagnostic accuracy for the assessment of sarcopenia when compared with reference imaging modalities, supporting its role as a feasible alternative in vulnerable populations [31]. In parallel, ultrasound-based monitoring of muscle mass has been linked to clinically relevant outcomes in critical care and oncology settings. Reductions in rectus femoris thickness or cross-sectional area have been associated with mortality, difficulty in weaning from mechanical ventilation, and prolonged rehabilitation in intensive care unit populations [6,7,18].

Nevertheless, although some studies have proposed threshold-based definitions of “significant” muscle wasting (e.g., >10% loss in rectus femoris thickness or cross-sectional area), associations with mortality have not been consistently observed, likely owing to limitations in sample size, follow-up duration, and study design [6,7].

The height-squared (m^2^)^–^-normalized muscle indices used in this study represent an adaptation of the skeletal muscle index (SMI) concept commonly applied to the assessment of appendicular muscle mass. Both the EWGSOP and the AWGS recommend adjusting muscle quantity for body size using height squared (m^2^) [25,26]. Indexing muscle measurements by height squared (m^2^) helps reduce anthropometric bias and allows more comparable assessments across individuals with different body sizes.

Compared with the rectus femoris, the literature on ultrasonographic assessment of upper-extremity muscles—particularly the biceps brachii—is more limited. Systematic reviews have consistently reported that the rectus femoris is the most frequently evaluated muscle in ultrasound-based sarcopenia research, whereas the biceps brachii has been examined in fewer studies [19,30]. Nevertheless, accumulating evidence suggests that upper-extremity muscle thickness may be associated with clinically relevant outcomes and may hold value for mortality assessment [32,33].

In addition, several prospective studies focusing on biceps brachii muscle thickness have demonstrated associations with mortality in older adults and palliative populations. However, these studies have almost exclusively relied on single-time-point measurements, providing only a static assessment of muscle status [8,21]. To our knowledge, no previous study has specifically evaluated short-term changes in biceps brachii muscle thickness as a prognostic marker.

Our findings extend the existing literature by moving beyond static anatomical measurements and emphasizing the prognostic relevance of dynamic muscle loss over a brief interval. The observed association between Δ BB MT-I and survival duration suggests that the rate of physiological deterioration, rather than a single snapshot of muscle mass, may better reflect the terminal disease trajectory.

Structural and functional differences between the rectus femoris (RF) and biceps brachii (BB) muscles may help explain the biological mechanisms underlying the observed findings. The rectus femoris plays a central role in lower-extremity extension and mobility and is particularly susceptible to early atrophy during immobilization—a phenomenon that has been well documented in clinical settings [6,7]. In contrast, the BB consists of a mixture of type I and type II fibers and is more frequently engaged in basic activities of daily living, such as eating, repositioning in bed, and assisted upper-limb movements. Age-related reductions in type II fiber area and their contribution to functional decline have been previously reported [22,24]. Continued intermittent use of the BB, even in terminally ill patients, may therefore allow short-term changes in BB muscle thickness to better reflect residual functional reserve and physiological frailty.

Although cross-sectional area (CSA) measurements theoretically provide a more comprehensive representation of muscle volume, ultrasound-based CSA assessment is inherently more operator-dependent, owing to variability in probe positioning, probe pressure, and visualization of muscle contours. In contrast, muscle thickness (MT) measurement relies on the linear distance between clearly defined fascial layers and generally offers higher repeatability [34,35]. In the present study, the finding that Δ BB MT-I remained independently associated with survival duration after adjustment for relevant clinical variables suggests that this parameter may represent a more reliable indicator of short-term, dynamic biological change.

The lack of statistically significant associations for traditional clinical indicators such as ECOG performance status, BMI, and NRS-2002 further suggests that static clinical and nutritional scores may have limited prognostic value in the terminal stage [30]. Serial muscle ultrasonography, by contrast, directly captures the rate of biological deterioration over time and may therefore provide complementary information that is not reflected in single-time-point clinical assessments [13].

The observed association between Δ BB MT-I and the number of days from the day-10 follow-up ultrasound to death suggests that this measure may reflect the short-term pace of physiological decline in palliative care patients.

An important methodological consideration in interpreting these findings is the potential for survivor or immortal-time bias introduced by the requirement to complete the 10-day ultrasound follow-up [36,37,38]. Because Δ-based analyses could only be performed in patients who survived and remained hospitalized until day 10, individuals with the most aggressive disease trajectories—those who died or were discharged earlier—were systematically excluded. This selection process may have resulted in preferential inclusion of patients with relatively longer early survival, potentially leading to an overestimation of the strength of the observed association between Δ BB MT-I and survival duration. Accordingly, the results should be interpreted as reflecting an association within a selected subgroup of terminally ill patients rather than as a definitive prognostic effect applicable to the entire palliative care population.

Although the study was not designed as a primary time-to-event analysis, the exploratory landmark Cox proportional hazards model yielded directionally consistent results, supporting the robustness of the observed association between short-term Δ BB MT-I loss and survival timing [38]. From a methodological perspective, translating this association into clinically actionable prediction will require prospective validation within a predefined analytical framework [39]. Future studies should incorporate a prespecified landmark time point (e.g., day-10 ultrasound), standardized measurement intervals, and inclusion of all eligible patients with appropriate censoring strategies.

Beyond estimating associations, such studies should also report prognostic performance metrics, including discrimination (e.g., C-statistics) and calibration, to assess the accuracy of Δ BB MT-I in predicting survival timing at the individual level [40]. Integrating serial ultrasound-derived muscle loss into multivariable prognostic models alongside clinical and laboratory parameters may ultimately facilitate the development of more reliable and clinically interpretable prognostic tools suitable for routine palliative care practice.

However, the observed relationship between Δ BB MT-I and mortality should not be interpreted as evidence of direct causality. Accelerated muscle loss in the terminal stage represents a phenotypic manifestation of multiple interrelated systemic processes, including tumor burden, chronic inflammation, catabolic metabolic responses, reduced nutritional intake, endocrine dysregulation, and prolonged immobilization, all of which converge in advanced malignancy [41,42]. These processes collectively reflect the severity and tempo of the underlying disease rather than a single isolated pathological pathway.

Accordingly, short-term muscle loss is unlikely to represent a direct cause of death but rather an integrated marker of the biological pace of disease progression. Within this context, our findings suggest that longitudinal monitoring of muscle loss may be informative for capturing the dynamics of physiological decline in terminal illness and for contextualizing changes in functional reserve over time.

From a clinical perspective, serial assessment of Δ BB MT-I may assist clinicians in contextualizing survival timing more realistically in terminal cancer patients. This information may support anticipatory care planning, the timing of discussions with patients and families, and alignment of treatment intensity with patient goals and preferences. While Δ BB MT-I should not be used as a standalone decision-making tool, it may complement existing clinical judgment in palliative care settings where prognostic uncertainty is substantial.

Most previous ultrasound-based studies have focused on baseline muscle mass, and relatively few investigations have evaluated mortality using Δ values derived from repeated measurements [43,44].

Studies incorporating Δ values have largely been conducted in intensive care unit (ICU) populations, where serial ultrasound assessments are primarily used to monitor clinical improvement or deterioration during critical illness. Rollinson et al. reported a significant decrease in rectus femoris cross-sectional area during ICU stay, with muscle loss continuing even after transfer to the general ward. These findings suggest that muscle wasting may not recover in parallel with apparent clinical improvement but instead may reflect the ongoing biological burden of disease [45]. Similarly, Kim et al. demonstrated that rehabilitation interventions could attenuate muscle loss in ICU patients [46], while Rosa Domingues et al. showed that skeletal muscle mass measured by ultrasound declined significantly during the first week of ICU admission, particularly among patients at nutritional risk [47].

In contrast, the present study was conducted in terminal-stage palliative cancer patients with irreversible clinical trajectories and focuses not only on the presence of muscle loss but also on the rate at which it occurs over a short interval and its association with the timing of mortality. Whereas most prior serial ultrasound studies have concentrated on lower-limb muscles—particularly the rectus femoris—to predict recovery or deterioration in critical illness [45], our study assessed the biceps brachii, a muscle that may better reflect residual functional reserve in terminally ill patients [32,33].

By demonstrating that Δ BB MT-I is associated not only with mortality but also with when death occurs, our findings help bridge ICU-based prognostic approaches with the palliative care context. Within this framework, the present results should be regarded as hypothesis-generating.

The single-center design, relatively small sample size, and exclusive focus on terminal malignant patients limit the generalizability of the findings. Nevertheless, the consistency of the observed associations across multiple analytical approaches supports the relevance of short-term muscle loss as a marker of disease trajectory in this specific clinical setting.

From a clinical perspective, serial ultrasound measurements and Δ BB MT-I may serve as complementary tools that provide additional prognostic information in terminal cancer patients. Although these measures are not sufficient on their own to guide treatment decisions, identification of rapid muscle loss may assist clinicians in communicating prognostic information more realistically, revisiting end-of-life care plans, and appropriately timing supportive interventions when feasible. However, the present study does not allow assessment of how such measurements could be directly integrated into formal clinical decision-making algorithms. Accordingly, prospective and intervention-focused studies are required before Δ BB MT-I can be incorporated into routine clinical practice. Integration of serial ultrasound-derived muscle loss into broader clinical workflows may ultimately strengthen decision-support processes in palliative care, particularly in settings characterized by high prognostic uncertainty.

### Limitations

This study has several limitations. First, owing to its single-center and retrospective design, the findings may be most applicable to the terminal malignant palliative care population treated at this institution, and generalizability is therefore limited. The sample size was relatively small; however, the rigorous application of predefined inclusion and exclusion criteria and the absence of missing ultrasound data enhance the internal consistency of the measurements.

All ultrasound assessments were performed by a single experienced operator, which likely improved measurement consistency but precluded evaluation of interobserver variability. Although this represents a potential limitation related to operator dependency, the use of standardized measurement protocols, clearly defined anatomical landmarks, fixed time points, and repeated measurements averaged across three acquisitions strengthens the reliability of the ultrasound data.

Ultrasound-based assessment of muscle mass is inherently subject to technique-related sources of variability, including probe pressure, probe angle, and interpretation of fascial borders. While methodological steps were taken to minimize these effects, muscle loss in terminal illness is also influenced by a wide range of clinical factors beyond structural muscle change, such as corticosteroid use, acute infections, systemic inflammation (e.g., C-reactive protein), endocrine responses, nutritional intake and support, and hydration status. Because these variables were not systematically recorded, they could not be incorporated as covariates, and residual confounding cannot be excluded. Accordingly, the observed association between Δ BB MT-I and survival duration should be interpreted as hypothesis-generating rather than as evidence of a causal relationship, reflecting the integrated biological burden of advanced disease rather than a single modifiable pathway.

Muscle measurements were expressed as indices normalized by height to improve comparability across individuals with different body sizes; however, other aspects of body composition—such as fat–muscle distribution, fluid shifts, and the presence of edema—were not directly assessed. These factors may have influenced ultrasound-derived measurements and could not be accounted for in the analyses.

Finally, because calculation of Δ required completion of the day-10 follow-up ultrasound, patients who died or were discharged before day 10 were not eligible for Δ-based analyses. This design-related selection may have introduced survivor or immortal-time bias and should be considered when interpreting the findings. Overall, the results should be viewed as preliminary, and larger prospective, multicenter studies with standardized measurement protocols and comprehensive covariate collection are needed to validate and extend these observations.

## 5. Conclusions

In this study, short-term muscle loss assessed by serial muscle ultrasonography in terminally ill cancer patients receiving palliative care—particularly Δ BB MT-I—was found to be associated with survival duration. These findings suggest that not only single-time-point muscle measurements, but also the rate of muscle loss over time, may provide clinically relevant information regarding survival timing. The lack of radiation exposure, bedside feasibility, and repeatability of ultrasonography make this approach particularly suitable for frail and immobile palliative care patients.

Overall, the results indicate that serial muscle ultrasonography may represent a dynamic biomarker candidate in palliative care practice, offering information beyond that provided by static clinical and nutritional parameters. However, larger prospective studies are required to validate these observations and to evaluate how such measurements could be integrated into clinical decision-making processes.

Future multicenter prospective investigations incorporating serial ultrasound assessments alongside inflammatory markers, nutritional intake, and treatment-related factors are warranted to further clarify the prognostic role of short-term muscle loss and to determine whether Δ BB MT-I can be incorporated into routine palliative care prognostic frameworks.

## Figures and Tables

**Table 1:** Demographic and Clinical Characteristics of the Patients (n = 87).

Variable	Value
Age (years), mean ± SD	74.6 ± 10.2
Male	47 (54.0%)
Female	40 (46.0%)
ECOG Score 0	6 (6.9%)
ECOG Score 1	6 (6.9%)
ECOG Score 2	26 (29.9%)
ECOG Score 3	27 (31.0%)
ECOG Score 4	22 (25.3%)
NRS-2002 Score 3	6 (6.9%)
NRS-2002 Score 4	16 (18.4%)
NRS-2002 Score 5	37 (42.5%)
NRS-2002 Score 6	28 (32.2%)
BMI: Normal (18.5–24.9)	48 (55.2%)
BMI: Underweight (<18.5)	17 (19.5%)
BMI: Overweight (25–29.9)	16 (18.4%)
BMI: Obese (≥30)	6 (6.9%)
Type of Malignancy	
Lung Cancer	19 (21.8%)
Head and Neck Cancers	14 (16%)
Genitourinary Cancers	18 (20.6%)
Gastrointestinal Cancers	29 (33.3%)
Malignant Melanoma	2 (2.2%)
Breast Cancer	4 (4.5%)
Soft Tissue Sarcoma	1 (1.1%)
One-year mortality	58 (66.7%)
One-year survivors	29 (33.3%)

Values are presented as mean ± standard deviation or n (%) where appropriate. BMI (Body Mass Index) was categorized according to the World Health Organization classification (Underweight: <18.5 kg/m^2^; Normal: 18.5–24.9 kg/m^2^; Overweight: 25–29.9 kg/m^2^; Obese: ≥30 kg/m^2^). ECOG (Eastern Cooperative Oncology Group) performance status was obtained from the clinical assessment at admission. NRS-2002 (Nutritional Risk Screening-2002) reflects nutritional risk.

**Table 2:** Ultrasound Muscle Measurements at Baseline and Day 10 (n = 87).

Parameter	Value
Baseline RF SFTT-I	0.242 (0.159–0.430)
Day 10 RF SFTT-I	0.201 (0.152–0.377)
Baseline RF MT-I	0.253 ± 0.089
Day 10 RF MT-I	0.224 ± 0.080
Baseline RF CSA-I	0.718 (0.596–0.875)
Day 10 RF CSA-I	0.651 (0.540–0.788)
Baseline BB SFTT-I	0.097 (0.059–0.149)
Day 10 BB SFTT-I	0.083 (0.057–0.144)
Baseline BB MT-I	0.505 ± 0.103
Day 10 BB MT-I	0.471 ± 0.106
Baseline BB CSA-I	1.442 (1.234–1.675)
Day 10 BB CSA-I	1.351 (1.101–1.577)

Normalized index values for muscle ultrasound measurements on baseline and day-10 follow-up are presented as mean ± standard deviation or median (IQR). For the RF (rectus femoris) and BB (biceps brachii) muscles, SFTT-I (subcutaneous fat thickness index), MT-I (muscle thickness index), and CSA-I (cross-sectional area index) were assessed. All index values were standardized by dividing each measurement by height squared (m^2^).

**Table 3:** Δ Values of Muscle Ultrasonography Parameters (n = 87).

Parameter	Min	Max	Median	IQR (25th–75th)
Δ RF SFTT-I	−0.3919	+0.0908	+0.052	0.011–0.121
Δ RF MT-I	−0.1374	+0.0480	+0.018	0.006–0.044
Δ RF CSA-I	−0.2630	+0.1217	+0.031	0.010–0.081
Δ BB SFTT-I	−0.0970	+0.0841	+0.009	0.003–0.033
Δ BB MT-I	−0.2148	+0.0391	+0.029	0.011–0.068
Δ BB CSA-I	−0.5453	+0.2704	+0.064	0.021–0.168

Δ indicates short-term muscle loss, calculated as baseline minus day-10 measurement and higher Δ indicates greater loss. Due to non-normal distribution, all parameters are presented as median (interquartile range, IQR). RF = rectus femoris; BB = biceps brachii; SFTT-I = subcutaneous fat thickness index; MT-I = muscle thickness index; CSA-I = cross-sectional area index. All index values were standardized by dividing each measurement by height squared (m^2^).

**Table 4:** Comparison of Baseline Clinical Variables and 10-Day Muscle Loss (Δ) Between Patients Who Died Within One Year and Those Who Survived (n = 87).

Variable	Died Within 1 Year (n = 58)	Survived ≥ 1 Year (n = 29)	*p*-Value
Age (years), mean ± SD	73.98 ± 10.44	75.89 ± 9.73	0.284
Sex (Male %)	54%	50%	0.527
BMI, mean ± SD	23.07 ± 5.80	21.54 ± 3.34	0.319
ECOG (median, IQR)	3 (2–3)	3 (2–4)	0.468
NRS-2002 (median, IQR)	5 (4–6)	5 (5–6)	0.171
Δ BB MT-I (median, IQR)	0.026 (0.010–0.070)	0.024 (0.006–0.071)	0.41
Δ BB CSA-I (median, IQR)	0.067 (0.025–0.184)	0.058 (0.017–0.152)	0.52
Δ BB SFTT-I (median, IQR)	0.011 (0.004–0.028)	0.009 (0.003–0.024)	0.46
Δ RF MT-I (median, IQR)	0.019 (0.008–0.045)	0.017 (0.007–0.042)	0.47
Δ RF CSA-I (median, IQR)	0.034 (0.012–0.081)	0.029 (0.009–0.075)	0.50
Δ RF SFTT-I (median, IQR)	0.022 (0.010–0.061)	0.019 (0.008–0.055)	0.44

All delta (Δ) parameters exhibited non-normal distributions and are therefore reported as median (IQR); between-group comparisons were performed using the Mann–Whitney U test. Δ indicates the short-term change in muscle measurements and reflects muscle loss over the 10-day period. For the BB (biceps brachii) muscle, SFTT-I (subcutaneous fat thickness index), MT-I (muscle thickness index), and CSA-I (cross-sectional area index) were measured. Index values were calculated by dividing each measurement by height squared (m^2^). ECOG: Eastern Cooperative Oncology Group; NRS-2002: Nutritional Risk Screening-2002.

**Table 5:** US Delta (Δ) Values and Survival Duration (Days from the day-10 follow-up ultrasound to death) (n = 58).

Parameter	Pearson r	95% CI (Pearson r)	*p* (Pearson)	Spearman r	*p* (Spearman)
Δ RF SFTT-I	0.191	−0.071 to 0.428	*p* = 0.151	0.131	*p* = 0.325
Δ RF MT-I	−0.139	−0.384 to 0.124	*p* = 0.299	−0.041	*p* = 0.762
Δ RF CSA-I	−0.251	−0.478 to 0.008	*p* = 0.057	−0.142	*p* = 0.286
Δ BB SFTT-I	0.042	−0.219 to 0.297	*p* = 0.755	0.009	*p* = 0.948
Δ BB MT-I	−0.437	−0.625 to −0.201	*p* = 0.0006 *	−0.240	*p* = 0.069
Δ BB CSA-I	−0.189	−0.426 to 0.073	*p* = 0.155	−0.092	*p* = 0.492

Δ indicates short-term muscle loss, calculated as baseline minus day-10 measurement and higher Δ indicates greater loss. For the RF (rectus femoris) and BB (biceps brachii) muscles, SFTT-I (subcutaneous fat thickness index), MT-I (muscle thickness index), and CSA-I (cross-sectional area index) were assessed. All index values were calculated by dividing each measurement by height squared (m^2^). Pearson correlation coefficients are reported with 95% confidence intervals calculated using Fisher’s z transformation (n = 58). * *p* < 0.05 was considered statistically significant.

**Table 6:** Multivariable Linear Regression Model for Survival Duration After Day-10 Ultrasound (n = 58).

Predictor	β	SE	95% CI	*p*-Value
Intercept	59.94	80.80	−98.4 to 218.3	0.458
Δ BB MT-I	−701.19	204.24	−1102.0 to −300.9	0.0006
Age	1.40	1.31	−1.17 to 3.97	0.285
Sex (Male)	−6.50	23.30	−52.2 to 39.2	0.780
ECOG	−6.79	9.29	−25.0 to 11.4	0.466
BMI	0.30	1.79	−3.21 to 3.81	0.867
NRS-2002	−20.37	14.67	−49.1 to 8.37	0.165

Δ indicates short-term muscle loss, calculated as baseline minus day-10 measurement and higher Δ indicates greater loss. BB MT-I: biceps brachii muscle thickness index; ECOG: Eastern Cooperative Oncology Group performance status; NRS-2002: Nutritional Risk Screening-2002; BMI: body mass index. This table presents a multivariable linear regression model evaluating the association between short-term change in BB MT-I over the 10-day interval and survival duration (days from the day-10 follow-up ultrasound to death), adjusted for age, sex, ECOG performance status, BMI, and NRS-2002 score.

## Data Availability

The data presented in this study are available from the corresponding author upon reasonable request.

## Supplementary Materials

The following supporting information can be downloaded at: https://www.mdpi.com/article/10.3390/medicina62020292/s1, Supplementary Table S1: Landmark Cox Proportional Hazards Analysis with Day-10 Ultrasound as Time Zero (n = 87); Supplementary Table S2. Sensitivity Analysis Adjusting for Baseline Ultrasound Measurement Day Multivariable Linear Regression Analysis for Survival Duration with Additional Adjustment for Baseline Ultrasound Measurement Day (n = 58).
