# Supplementary material for: Short-Term Biceps Muscle Wasting Assessed by Serial Ultrasound as a Predictor of Survival Duration in Terminally Ill Cancer Patients: A Retrospective Cohort Study
Source: Medicina (Kaunas). 2026 Feb 1;62(2):292. doi: 10.3390/medicina62020292 (PMC12941632; doi:10.3390/medicina62020292)
Supplement: Supplementary file 1 [file medicina-62-00292-s001.zip › medicina-4068227-supplementary.pdf]

Supplementary Table S1. Landmark Cox Proportional Hazards Analysis with Day-10 Ultrasound as Time Zero (n = 87)

Predictor	HR	95% CI	p
**Δ BB MT-I (per 0.10-unit change)**	1.60	1.04 – 2.44	0.033
**Age (years)**	1.001	0.968 – 1.036	0.939
**Male sex**	0.790	0.453 – 1.376	0.405
**ECOG**	0.958	0.763 – 1.203	0.713
**BMI (kg/m²)**	1.042	0.997 – 1.089	0.070
**NRS-2002**	0.856	0.576 – 1.273	0.443

Δ BB MT-I indicates short-term muscle loss in the biceps brachii muscle thickness index between baseline and day-10 measurements. A landmark Cox proportional hazards model was applied with the day-10 follow-up ultrasound defined as time zero. Survival time was calculated as the number of days from the day-10 follow-up ultrasound to death, with patients who were alive at one year censored at 365 days. Hazard ratios for Δ BB MT-I are expressed per 0.10-unit increase using rescaling based on the log-linear property of the Cox proportional hazards model. All models were adjusted for age, sex, Eastern Cooperative Oncology Group (ECOG) performance status, body mass index (BMI), and Nutritional Risk Screening-2002 (NRS-2002) score. HRs are presented with 95% confidence intervals (CI). All p-values are two-sided, and $p < 0.05$ was considered statistically significant.

Supplementary Table S2. Sensitivity Analysis Adjusting for Baseline Ultrasound Measurement Day

Multivariable Linear Regression Analysis for Survival Duration with Additional Adjustment for Baseline Ultrasound Measurement Day (n = 58)

Predictor	β (days)	SE	95% CI	p-value
**Δ BB MT-I**	−696.6	211.8	−1121.2 to −272.0	0.0018
**Baseline ultrasound day (1–3)**	1.82	18.4	−35.2 to 38.8	0.92
**Age (years)**	1.39	1.32	−1.20 to 3.98	0.29
**Sex (male)**	−6.43	23.5	−52.7 to 39.8	0.78
**ECOG**	−6.75	9.34	−25.1 to 11.6	0.47
**BMI (kg/m²)**	0.31	1.80	−3.23 to 3.85	0.87
**NRS-2002**	−20.1	14.7	−49.0 to 8.8	0.17

Δ BB MT-I indicates the short-term change in biceps brachii muscle thickness index. Baseline ultrasound day refers to the timing of the initial ultrasound assessment relative to hospital admission (day 1–3). Survival duration was defined as the number of days from the day-10 follow-up ultrasound to death among patients who died within one year. All models were adjusted for age, sex, Eastern Cooperative Oncology Group (ECOG) performance status, body mass index (BMI), and Nutritional Risk Screening-2002 (NRS-2002) score. β coefficients represent the change in survival duration (days) per unit change in the predictor. The inclusion of baseline ultrasound measurement day did not materially alter the association between Δ BB MT-I and survival duration.

https://doi.org/10.3390/xxxxx
